# Refinement of K[HgI_3_]·H_2_O using non-spherical atomic form factors

**DOI:** 10.1107/S2056989021005582

**Published:** 2021-06-04

**Authors:** Misael Chocolatl Torres, Sylvain Bernès, Ulises Salazar Kuri

**Affiliations:** a Instituto de Física, Benemérita Universidad Autónoma de Puebla, 72570 Puebla, Pue., Mexico

**Keywords:** crystal structure, redetermination, atomic form factors, NoSpherA2

## Abstract

The crystal structure model for potassium tri­iodido­mercurate(II) monohydrate, K[HgI_3_]·H_2_O, based on single-crystal data, was reported 50 years ago [Nyqvist & Johansson (1971 ▸). *Acta Chem. Scand*. **25**, 1615–1629]. We have now redetermined this structure with X-ray diffraction data at 0.70 Å resolution collected at 153 K using Ag *K*α radiation. Combined quantum mechanical methods (*ORCA*) and computation of non-spherical scattering form factors (*NoSpherA2*) allowed the refinement of the shape of the water mol­ecule with anisotropic H atoms, despite the presence of heavy elements in the crystal. The refined shape of the water mol­ecule *via* this Hirshfeld refinement is close to that determined for liquid water by neutron diffraction experiments. Moreover, the Laplacian of the electron density clearly shows how electron density accumulates along the O—H σ-valence bonds in the water mol­ecule.

## Abstract 2

The structure of K[HgI_3_]·H_2_O was redetermined at 0.70 Å resolution, and its conventional refinement is compared to a refinement using non-spherical atomic form factors.

## Chemical context   

It is well known that the ‘independent atom model’ (IAM), universally implemented in mainstream X-ray crystallography software, has the drawback of affording insufficient crystal structure models. Given that a spherical distribution of electron density around each atom is assumed, for example, by using the Cromer–Mann parameterization of the non-dispersive part of the form factors, any density involved in bonds, lone pairs and inter­molecular charge transfer is completely ignored. In this context, satisfactory structure models can be obtained only on the basis of neutron diffraction data. An extreme case of discrepancy between results obtained with both radiations is the O—H bond length for the hydroxyl group in alcohols and water, which is underestimated by *ca* 20% by X-rays. However, neutron diffraction facilities are scarce, and even non-existent in underdeveloped countries. As a matter of fact, only 0.2% of the structures currently deposited in the CSD originate from neutron diffraction studies (Groom *et al.*, 2016 ▸).

Within many approaches available to overcome this issue, the ‘Hirshfeld atom refinement’ (HAR; Capelli *et al.*, 2014 ▸) strategy is gaining popularity. After calculating a mol­ecular wave function for a structural model (not necessarily limited to the asymmetric unit), the electronic density functions of the so-called Hirshfeld atoms are extracted through a partitioning process (Hirshfeld, 1977 ▸), and eventually Fourier transformed, to afford non-spherical scattering factors for each individual atom in the real space and each reflection in the reciprocal space. More accurate structure factors can then be calculated during a least-squares refinement, and the full process can be iterated until convergence.

A user-friendly implementation of HAR has been recently released with *OLEX2* (version 1.3) and is fully inter­faced with the *olex2.refine* least-squares engine (Kleemiss *et al.*, 2021 ▸). This new tool, coined as *NoSpherA2* (pronounced ‘Nosferatu’), is virtually universal since any element can be present in the structure. Moreover, the structure can be disordered, with atoms in special positions, squeezed with a solvent mask, or can include restrained parts. Twinned crystals can also be handled in the same way as single crystals, by computing a single wave function for each twin component. Finally, data resolution is not a concern, as long as atomic resolution is achieved [*d*_min_ = 0.84 Å, corresponding to (sin θ/λ)_max_ = 0.6 Å^−1^]. At worst, a data set with no information at all about aspherical local densities would give a Hirshfeld refinement close to that obtained with Cromer–Mann form factors.

So far, HAR has been used mainly for organic compounds, for at least two reasons. Many accurate orbital basis sets are available for light elements and, more significantly, this class of mol­ecules is the most inter­esting one for such refinements: organic compounds include a large variety of chemical bonds (*σ*, *π*, aromatic, 2*c*–3*e* bonds, *etc*.) and heteroatoms frequently bear electron lone pairs. The structural model obtained *via* HAR is thus expected to be greatly improved compared to that derived from a traditional refinement with spherical densities.

We used *NoSpherA2* to refine the crystal structure of a material including both heavy and light elements, with the aim of assessing whether a non-spherical refinement is suitable and useful for such materials. The matter has been already studied for challenging compounds, namely transition-metal hydrides (Woińska *et al.*, 2021 ▸; Kleemiss *et al.*, 2021 ▸), and is now extended to an iodido­mercurate hydrate, K[HgI_3_]·H_2_O.

## Structural commentary   

**Figure 1:**
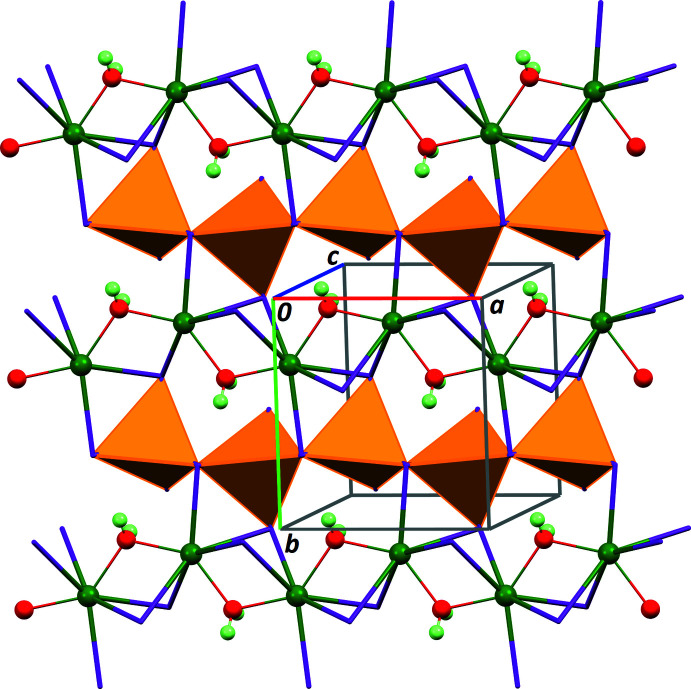
Part of the crystal structure of the title compound. Colour code: orange = [HgI_4_] tetra­hedra, purple = I, green = K, red = O, pale green = H.

The crystal structure of potassium tri­iodido­mercurate(II) monohydrate, K[HgI_3_]·H_2_O, was reported 50 years ago, using data collected on a Philips–Norelco PAILRED diffractometer, with monochromatized Mo *K* radiation (1542 reflections in the 0*kl*–10*kl* half-sphere; *R* = 0.081 for an anisotropic model omitting H atoms; Nyqvist & Johansson, 1971 ▸). The powder diffraction pattern is also deposited in the PDF-2 database, with reference PDF 00-027-0415 (Gates-Rector & Blanton, 2019 ▸). Using low-temperature data collected with Ag *K*α radiation, we now obtained the same structure at 0.70 Å resolution in the same space group, *Pna*2_1_ (Fig. 1 ▸ and Table 1 ▸). The Hg atoms form distorted [HgI_4_] tetra­hedra sharing one corner and giving a chain structure along the *a-*axis direction. Water mol­ecules bridge K^+^ cations and are sandwiched between these chains, at normal distances, K—OH_2_ ≃ 2.75 Å. The cations are seven-coordinate, a common coordination number for K^+^, characterized by its large ionic radius. The three-dimensional structure is completed by K^+^ cations bridg­ing [HgI_4_] tetra­hedra in neighbouring chains. The water mol­ecules are oriented in such a way that O—H⋯I hydrogen bonds are formed with two I atoms on the edge of an [HgI_4_] tetra­hedron.

Although H atoms were visible in a difference-Fourier map, the IAM refinement carried out with *SHELXL* (Sheldrick, 2015*b* ▸) gave an odd shape for the water mol­ecule. Hydroxyl O—H groups were then restrained to have the same bond lengths with an effective standard deviation of 0.04 Å. Rigid bond restraints with a standard deviation of 0.008 Å for 1,2 and 1,3 distances in the K/O1/H1*a*/H1*b* fragment were also applied. Both O—H bond lengths in the water mol­ecule converged to 0.84 (11) Å, and the H—O—H angle was too acute, 87 (10)°. Moreover, isotropic displacement parameters for the H1*a* and H1*b* atoms were unbalanced, 0.06 (5) and 0.18 (9) Å^2^, respectively. For this preliminary refinement, hydrogen bonds were determined with large uncertainties for O—H⋯I angles, 160 (12) and 159 (26)°.

**Figure 2:**
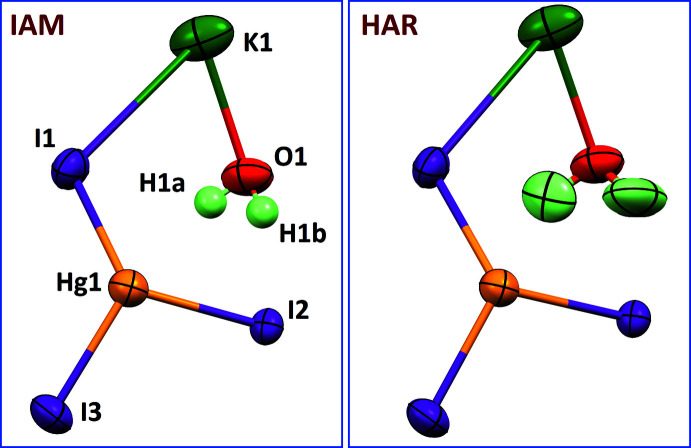
Ellipsoid plots of the asymmetric unit for the IAM (left) and HAR (right) models, with displacement ellipsoids at the 85% probability level. For the IAM refinement, isotropic H atoms are shown as spheres of arbitrary radius, while anisotropic H atoms in the HAR panel are shown with their refined ADPs.

With the hope of improving the shape of the water mol­ecule, a non-spherical refinement was carried out using the *SHELXL* model as a starting point. The wave functions were calculated using *ORCA* with the two-component relativistic basis set x2c-TZVPP and the generalized gradient approximation PBE functional (Neese, 2018 ▸). The least-squares refinements were then carried out with *olex2.refine* (Bourhis *et al.*, 2015 ▸), while keeping the same restraints as for the *SHELXL* refinement. For the final calculation of non-spherical form factors with *NoSpherA2*, a neutral dimeric cluster [KHgI_3_·H_2_O]_2_ was used as a structure model, in order to take into account O—H⋯I hydrogen bonds. The final refinement was done with *olex2.refine* (Table 1 ▸), and a comparison of the asymmetric units for the IAM and HAR refinements is given in Fig. 2 ▸.

The heavy part of the structure is almost unchanged after HAR, as expected. When comparing bonds lengths and angles, the largest difference is observed for the K—O bonds, with a shift of 0.006 Å; for bond angles, the largest difference between the two refinements is 0.25° for the angle K1—O1—K1^i^ [symmetry code: (i) *x* + 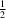, −*y* + 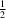, *z*]. Moreover, uncertainties for bond lengths and angles are systematically improved with HAR. Likewise, displacement parameters for Hg, I and K atoms are almost unaffected after using non-spherical form factors. In contrast, the water mol­ecule clearly displays a more accurate shape. Bond lengths for the O—H groups are 1.07 (6) and 1.11 (7) Å for the HAR model, with an H—O—H angle of 107 (8)°. For liquid water, neutron diffraction experiments afforded O—H = 0.970 ± 0.005 Å and H—O—H = 106.1 ± 1.8° (Ichikawa *et al.*, 1991 ▸; Milovanović *et al.*, 2020 ▸). These dimensions are also consistent with the shape previously described for a water mol­ecule bridging two K^+^ cations in a potassium aryl­oxide aggregate characterized by neutron diffraction at 100 K: O—H = 0.963 (16)–1.009 (16) Å and H—O—H = 108.0 (13)° (Morris *et al.*, 2007 ▸). It was possible to refine anisotropic displacement parameters for the H atoms, although it was necessary to use rigid bond restraints for the K—OH_2_ group, in order to avoid non-positive definite H atoms. In the final model, displacement ellipsoids for H atoms are well balanced (Fig. 2 ▸).

**Figure 3:**
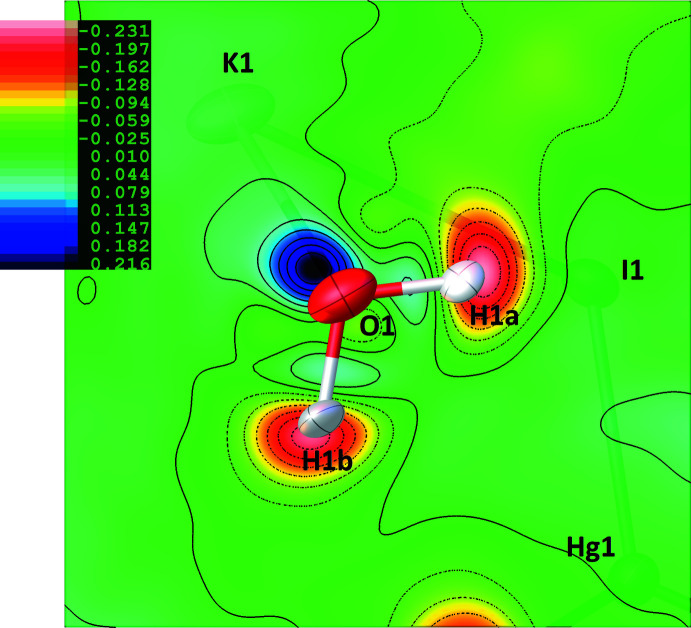
HAR – IAM dynamic deformation density map in the plane of the water mol­ecule. Isolevel contours for positive density (e^−^/A^3^) are displayed as solid lines with the map coloured blue, while isolevel contours for negative density are displayed as dashed lines, with the map coloured red. The map was plotted with *OLEX2* (Dolomanov *et al.*, 2009 ▸).

**Figure 4:**
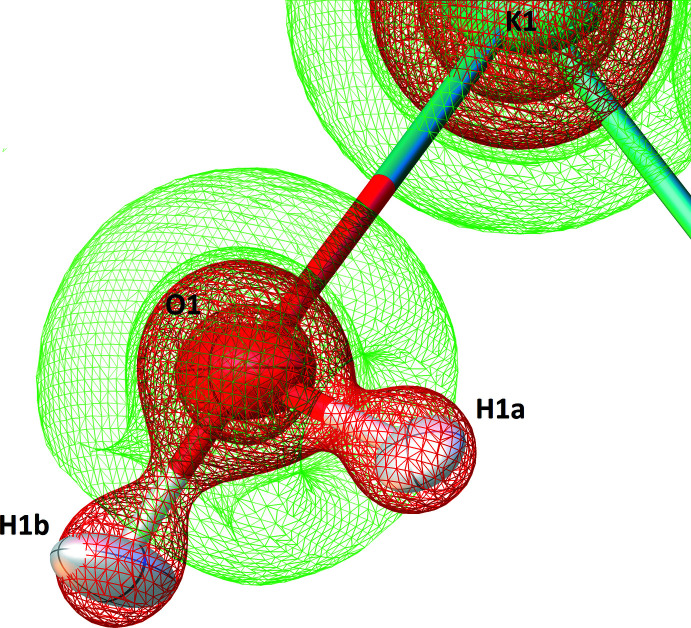
Three-dimensional wire map of the Laplacian of the electron density in the vicinity of the water mol­ecule, at ±0.25 e^−^/Å^5^ level. The positive isosurfaces (green) show where electron density depletion occurs (valence-atomic orbital regions), while negative isosurfaces (red) show regions where electron density accumulates (bonding-electrons energy densities). The map was calculated on a 0.05 Å grid in real space and was generated with *OLEX2* (Dolomanov *et al.*, 2009 ▸).

The final residual map is featureless, but the deformation density map in the water mol­ecule vicinity is insightful (Fig. 3 ▸). A positive density close to the O atom reflects the presence of electron lone pairs, while a negative density centred on the H-atom sites indicates the positively charged character of the H atoms, as a consequence of the difference of electronegativity with the O atom. A diffuse positive density is even visible at the midpoint of the O—H bonds, related to the contribution of the covalent *σ*-bonds to non-spherical densities. Beyond features observed for the water mol­ecule, the deformation map is flat, confirming that a Hirshfeld refinement adds very little to the conventional IAM approximation in those parts. Finally, the Laplacian of the electron density, 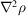, also shows expected features. Electronic density is locally concentrated over the attractive covalent O—H *σ*-bonds in the water mol­ecule (Fig. 4 ▸), while heavy atoms display 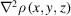 iso­surfaces with spherical symmetry.

## Discussion and conclusions   

Regarding the crystal structure refinement, the drop for resid­uals *R*_1_ and *wR*_2_ is marginal with a HAR compared to a IAM refinement with *SHELXL*, at any resolution, since the structure-factor amplitudes are dominated by the contribution of heavy scatterers, Hg and I. However, in the present case, diffraction data contain information about the non-sphericity of the form factors for the O and H atoms, warranting a HAR. Given that computational cost associated with the calculation of the wave function increases drastically for large mol­ecular systems or large clusters of mol­ecules, HAR may prove challenging to implement as a day-to-day routine, as long as desktop computers are used for structure refinements. However, the refinement reported here shows that an alternative would be to perform refinements through a hybrid IAM/HAR strategy, with structure factors including conventional spherical form factors for heavy atoms, and non-spherical form factors for light atoms. Obviously, this may not apply to large organic systems, like proteins, unless super-computing is involved (Capelli *et al.*, 2014 ▸).

## Synthesis and crystallization   

**Caution!!** Any mercury compound poses potential health risks; appropriate safety precautions and disposal procedures must be taken to handle the complexes here reported.

The compound under study was obtained as a by-product during the synthesis of Ag_2_[HgI_4_]. A procedure to obtain Ag_2_[HgI_4_] single crystals involves the near saturation of K_2_[HgI_4_] with HgI_2_ and AgI in an aqueous medium (Browall *et al.*, 1974 ▸). Potassium tetra­iodo­mercurate(II), commonly known as Nessler reagent, was obtained by dissolving 2.603 g of KI and 3.574 g of HgI_2_ in an aqueous medium, following the reaction: HgI_2_ + 2 KI → K_2_[HgI_4_]. The resulting solution was nearly saturated with HgI_2_ and subsequently with AgI. The solution was kept under constant stirring for 30 min at 323 K. After that, the solution was stored in 50 ml plastic tubes in complete darkness for one month.

The crystals obtained were washed with a 2 *M* solution of K_2_[HgI_4_] and distilled water. Since the process for the preparation of these compounds contains the precursors HgI_2_ and KI in an aqueous medium, this also favours the crystallization of K[HgI_3_]·H_2_O within a temperature range of 273–353 K (Sieskind *et al.*, 1998 ▸). One small crystal of K[HgI_3_]·H_2_O recovered from such a crystallization was used for the present study.

## Refinement details   

Crystal data, data collection, and structure refinement details for the last least-squares cycle of *olex2.refine* are summarized in Table 1 ▸. All atoms were refined anisotropically. In the water mol­ecule, O—H bonds were restrained to have the same length, with a standard deviation of 0.04 Å. Rigid bond restraints with a standard deviation of 0.008 Å for 1,2 and 1,3 distances in the K—OH_2_ fragment were also applied.

## Supplementary Material

Crystal structure: contains datablock(s) I, global. DOI: 10.1107/S2056989021005582/wm5609sup1.cif

Structure factors: contains datablock(s) I. DOI: 10.1107/S2056989021005582/wm5609Isup2.hkl

CCDC reference: 2087087

Additional supporting information:  crystallographic information; 3D view; checkCIF report

## Figures and Tables

**Table 1:** Experimental details

Crystal data	
Chemical formula	K[HgI_3_]·H_2_O
*M*_r_	638.41
Crystal system, space group	Orthorhombic, *P* *n* *a*2_1_
Temperature (K)	153
*a*, *b*, *c* (Å)	8.5810 (2), 9.2648 (3), 11.4073 (4)
*V* (Å^3^)	906.89 (5)
*Z*	4
Radiation type	Ag *K*α, λ = 0.56083 Å
μ (mm^−1^)	14.87
Crystal size (mm)	0.06 × 0.05 × 0.03

Data collection	
Diffractometer	Stoe Stadivari
Absorption correction	Multi-scan (*X-AREA*; Stoe & Cie, 2019 ▸)
*T*_min_, *T*_max_	0.064, 0.132
No. of measured, independent and observed [*I* > 2σ(*I*)] reflections	29699, 2720, 2179
*R*_int_	0.070
(sin θ/λ)_max_ (Å^−1^)	0.714

Refinement	
*R*[*F*^2^ > 2σ(*F* ^2^)], *wR*(*F* ^2^), *S*	0.021, 0.038, 0.87
No. of reflections	2720
No. of parameters	74
No. of restraints	21
H-atom treatment	All H-atom parameters refined
Δρ_max_, Δρ_min_ (e Å^−3^)	1.17, −1.26
Absolute structure	Flack (1983 ▸)
Absolute structure parameter	0.033 (11)

Computer programs: *X-AREA* (Stoe & Cie, 2019 ▸), *SHELXT2018/2* (Sheldrick, 2015*a* ▸), *olex2.refine 1.3* (Bourhis *et al.*, 2015 ▸), *OLEX2* (Dolomanov *et al.*, 2009 ▸), *Mercury* (Macrae *et al.*, 2020 ▸) and *publCIF* (Westrip, 2010 ▸).

## supplementary crystallographic information

### Crystal data

**Table:**

K[HgI_3_]·H_2_O	*D*_x_ = 4.676 Mg m^−^^3^
*M**_r_* = 638.41	Ag *K*α radiation, λ = 0.56083 Å
Orthorhombic, *P**n**a*2_1_	Cell parameters from 25947 reflections
*a* = 8.5810 (2) Å	θ = 2.2–30.8°
*b* = 9.2648 (3) Å	µ = 14.87 mm^−^^1^
*c* = 11.4073 (4) Å	*T* = 153 K
*V* = 906.89 (5) Å^3^	Block, colourless
*Z* = 4	0.06 × 0.05 × 0.03 mm
*F*(000) = 1072	

### Data collection

**Table:**

Stoe Stadivari diffractometer	2720 independent reflections
Radiation source: Sealed X-ray tube, Axo Astix-f Microfocus source	2179 reflections with *I* > 2σ(*I*)
Graded multilayer mirror monochromator	*R*_int_ = 0.070
Detector resolution: 5.81 pixels mm^-1^	θ_max_ = 23.6°, θ_min_ = 2.2°
ω scans	*h* = −12→12
Absorption correction: multi-scan (X-AREA; Stoe & Cie, 2019)	*k* = −13→13
*T*_min_ = 0.064, *T*_max_ = 0.132	*l* = −16→16
29699 measured reflections	

### Refinement

**Table:**

Refinement on *F*^2^	Hydrogen site location: difference Fourier map
Least-squares matrix: full	All H-atom parameters refined
*R*[*F*^2^ > 2σ(*F*^2^)] = 0.021	*w* = 1/[σ^2^(*F*_o_^2^) + (0.0157*P*)^2^] where *P* = (*F*_o_^2^ + 2*F*_c_^2^)/3
*wR*(*F*^2^) = 0.038	(Δ/σ)_max_ = 0.0003
*S* = 0.87	Δρ_max_ = 1.17 e Å^−^^3^
2720 reflections	Δρ_min_ = −1.25 e Å^−^^3^
74 parameters	Extinction correction: SHELXL2018/3 (Sheldrick 2015b), Fc^*^=kFc[1+0.001xFc^2^λ^3^/sin(2θ)]^-1/4^
21 restraints	Extinction coefficient: 0.00015 (5)
0 constraints	Absolute structure: Flack (1983)
Primary atom site location: dual	Absolute structure parameter: 0.033 (11)
Secondary atom site location: difference Fourier map	

### Special details

**Table:**

Refinement. Refinement using NoSpherA2, an implementation of NOn-SPHERical Atom-form-factors in Olex2. Please cite: F. Kleemiss *et al.* DOI 10.1039/D0SC05526C - 2020 NoSpherA2 implementation of HAR makes use of tailor-made aspherical atomic form factors calculated on-the-fly from a Hirshfeld-partitioned electron density (ED) - not from spherical-atom form factors.The ED is calculated from a gaussian basis set single determinant SCF wavefunction - either Hartree-Fock or DFT using selected funtionals - for a fragment of the crystal. This fregment can be embedded in an electrostatic crystal field by employing cluster charges. The following options were used: SOFTWARE: ORCA PARTITIONING: NoSpherA2 INT ACCURACY: High METHOD: PBE BASIS SET: x2c-TZVPP CHARGE: 0 MULTIPLICITY: 1 RELATIVISTIC: DKH2 DATE: 2021-04-12_22-39-08

### Fractional atomic coordinates and isotropic or equivalent isotropic displacement parameters (Å^2^)

**Table:**

	*x*	*y*	*z*	*U*_iso_*/*U*_eq_	
Hg1	0.25561 (3)	0.70101 (2)	0.500694 (18)	0.02464 (6)	
I1	0.25904 (7)	0.42035 (5)	0.56854 (3)	0.02692 (9)	
I2	0.49597 (5)	0.77133 (3)	0.33996 (3)	0.01831 (7)	
I3	0.23628 (7)	0.92253 (5)	0.65827 (3)	0.02791 (10)	
K1	0.44530 (17)	0.15898 (17)	0.3610 (2)	0.0413 (4)	
O1	0.6331 (5)	0.4017 (5)	0.3698 (5)	0.0321 (11)	
H1a	0.634 (12)	0.437 (11)	0.459 (6)	0.034 (16)	
H1b	0.607 (14)	0.498 (9)	0.316 (9)	0.06 (2)	

### Atomic displacement parameters (Å^2^)

**Table:**

	*U*^11^	*U*^22^	*U*^33^	*U*^12^	*U*^13^	*U*^23^
Hg1	0.02658 (10)	0.02249 (10)	0.02484 (11)	−0.00072 (11)	0.00067 (13)	−0.00180 (11)
I1	0.0331 (2)	0.0247 (2)	0.02295 (19)	−0.0019 (2)	−0.0010 (2)	0.00594 (16)
I2	0.01527 (15)	0.02130 (15)	0.01837 (17)	0.00014 (15)	−0.00029 (16)	0.0012 (2)
I3	0.0337 (2)	0.0264 (2)	0.0236 (2)	0.0034 (2)	−0.0026 (3)	−0.00697 (17)
K1	0.0251 (7)	0.0278 (7)	0.0711 (13)	0.0039 (5)	−0.0017 (8)	0.0034 (9)
O1	0.030 (2)	0.020 (2)	0.047 (3)	0.0026 (18)	0.002 (2)	−0.002 (2)
H1a	0.02 (4)	0.04 (2)	0.047 (10)	0.007 (15)	0.002 (7)	−0.005 (5)
H1b	0.10 (5)	0.021 (16)	0.05 (2)	0.001 (11)	−0.017 (16)	−0.003 (8)

### Geometric parameters (Å, º)

**Table:**

Hg1—I1	2.7131 (5)	I3—K1^iv^	3.659 (2)
Hg1—I2	2.8356 (5)	I3—K1^v^	3.7067 (19)
Hg1—I2^i^	2.8968 (5)	K1—O1^ii^	2.739 (5)
Hg1—I3	2.7333 (5)	K1—O1	2.769 (5)
I1—K1^ii^	3.6595 (19)	O1—H1a	1.07 (6)
I1—K1	3.745 (2)	O1—H1b	1.11 (7)
I2—K1^iii^	3.6257 (16)		
			
I3—Hg1—I1	122.189 (16)	I2^vii^—K1—I1	137.19 (6)
I2—Hg1—I1	113.375 (16)	I1^viii^—K1—I1	92.00 (5)
I3—Hg1—I2	107.267 (15)	I3^ix^—K1—I1	148.42 (5)
I2^i^—Hg1—I1	105.885 (15)	I3^x^—K1—I1	77.82 (3)
I3—Hg1—I2^i^	107.648 (15)	O1^ii^—K1—I2^vii^	85.19 (10)
I2—Hg1—I2^i^	97.460 (14)	O1—K1—I2^vii^	137.50 (10)
K1^ii^—I1—Hg1	90.00 (3)	O1^ii^—K1—I1^viii^	130.89 (15)
K1—I1—Hg1	116.37 (3)	O1—K1—I1^viii^	73.23 (12)
K1^iii^—I2—Hg1	95.60 (3)	O1^ii^—K1—I3^ix^	135.32 (15)
K1^iii^—I2—Hg1^vi^	87.86 (3)	O1—K1—I3^ix^	75.86 (11)
K1^iv^—I3—Hg1	102.44 (3)	O1^ii^—K1—I3^x^	75.34 (12)
K1^v^—I3—Hg1	86.63 (3)	O1—K1—I3^x^	74.46 (12)
K1—I1—K1^ii^	77.01 (4)	O1^ii^—K1—I1	72.08 (12)
K1^iv^—I3—K1^v^	77.50 (4)	O1—K1—I1	72.56 (11)
Hg1—I2—Hg1^vi^	99.816 (14)	K1—O1—K1^viii^	113.65 (15)
I2^vii^—K1—I1^viii^	75.86 (3)	O1^ii^—K1—O1	137.29 (12)
I3^ix^—K1—I2^vii^	70.37 (3)	H1a—O1—K1^viii^	95 (5)
I3^ix^—K1—I1^viii^	79.52 (3)	H1a—O1—K1	106 (6)
I3^x^—K1—I2^vii^	131.42 (6)	H1b—O1—K1^viii^	110 (6)
I3^x^—K1—I1^viii^	147.69 (5)	H1b—O1—K1	121 (6)
I3^ix^—K1—I3^x^	93.18 (5)	H1b—O1—H1a	107 (8)

Symmetry codes: (i) *x*−1/2, −*y*+3/2, *z*; (ii) *x*−1/2, −*y*+1/2, *z*; (iii) *x*, *y*+1, *z*; (iv) −*x*+1, −*y*+1, *z*+1/2; (v) −*x*+1/2, *y*+1/2, *z*+1/2; (vi) *x*+1/2, −*y*+3/2, *z*; (vii) *x*, *y*−1, *z*; (viii) *x*+1/2, −*y*+1/2, *z*; (ix) −*x*+1, −*y*+1, *z*−1/2; (x) −*x*+1/2, *y*−1/2, *z*−1/2.

### Hydrogen-bond geometry (Å, º)

**Table:**

*D*—H···*A*	*D*—H	H···*A*	*D*···*A*	*D*—H···*A*
O1—H1*a*···I3^vi^	1.07	2.76	3.777 (6)	158
O1—H1*b*···I2	1.11	2.72	3.637 (5)	140

Symmetry code: (vi) *x*+1/2, −*y*+3/2, *z*.
